# Role and Techniques of Splenic Angioembolization in the Management of High-Grade (AAST III-V) Blunt Splenic Trauma: A Systematic Review

**DOI:** 10.7759/cureus.105221

**Published:** 2026-03-14

**Authors:** Murhaf Assaf, Abdulaziz Alsamani Abdullah Omar, Kiranjot Kaur, Beshr Mosa Basha, Shashwat Shetty, Anwar Al-Kassar, Jamal Khan

**Affiliations:** 1 General Surgery, Princess of Wales Hospital, Cwm Taf Morgannwg University Health Board, Bridgend, GBR; 2 General Surgery, Prince Othman Digna Teaching Hospital, Port Sudan, SDN; 3 US Navy, United States Military, Great Lakes, USA; 4 Clinical Research, Arizona State University, Tempe, USA; 5 Medicine, Shri B. M. Patil Medical College, Bijapur, IND; 6 General Surgery, Dr. Sulaiman Al Habib Medical Group, Khobar, SAU; 7 Orthopaedics, Hillingdon Hospital, Uxbridge, GBR; 8 Vascular Surgery, Countess of Chester Hospital, Chester, GBR; 9 Internal Medicine, Pakistan Institute of Medical Sciences, Islamabad, PAK

**Keywords:** aast grade iii-v, blunt splenic trauma, non-operative management, proximal embolization, splenic artery embolization

## Abstract

Blunt splenic trauma is the most common solid organ injury in abdominal trauma, with high-grade (American Association for the Surgery of Trauma (AAST) III-V) injuries carrying increased risk of hemorrhage and failure of non-operative management (NOM). Splenic artery embolization (SAE) has emerged as a key adjunct for hemodynamically stable patients, aiming to achieve hemostasis while preserving splenic function. This systematic review evaluated SAE outcomes in high-grade blunt splenic trauma, focusing on technical success, splenic salvage, re-bleeding, complications, delayed splenectomy, and mortality. Five retrospective cohort studies, including 600 adults, were analyzed. SAE demonstrated high technical success (92-100%) and splenic salvage rates (>90%). Proximal and distal embolization achieved comparable salvage rates; distal embolization was associated with longer fluoroscopy times and occasional re-bleeding, while combined techniques showed higher abscess formation. Overall mortality was low, and SAE facilitated preservation of splenic function, reducing the need for delayed splenectomy. Limitations include retrospective design, moderate-to-serious risk of bias, and limited long-term immunologic data. SAE is a safe and effective adjunct to NOM in high-grade blunt splenic trauma, with technique selection influencing complication profiles. Prospective studies are needed to optimize embolization strategies and evaluate long-term outcomes.

## Introduction and background

Blunt splenic trauma is the most frequently injured solid organ in blunt abdominal trauma and represents a significant proportion of emergency surgical admissions worldwide [1]. The spleen is highly vascular and particularly vulnerable to deceleration and compressive forces, making it susceptible to injury in road traffic collisions, falls, and assaults. Historically, splenectomy was considered the standard treatment for splenic injury; however, recognition of the spleen’s immunological importance, particularly its role in protection against encapsulated organisms, has shifted management toward splenic preservation whenever feasible [2]. Advances in trauma systems, cross-sectional imaging, and critical care have facilitated the evolution of non-operative management (NOM) as the preferred approach in hemodynamically stable patients. The American Association for the Surgery of Trauma (AAST) Organ Injury Scale provides standardized grading from I to V based on parenchymal disruption and vascular involvement. While low-grade injuries (I-II) are associated with excellent outcomes under observation alone, high-grade injuries (III-V) carry a significantly increased risk of delayed hemorrhage, pseudoaneurysm formation, arteriovenous fistula, and failure of NOM [3]. Reported failure rates of NOM in high-grade injuries range between 10% and 20% in the absence of adjunctive intervention [4]. Consequently, splenic artery embolization (SAE) has emerged as a critical adjunct in patients with high-grade injuries who remain hemodynamically stable but demonstrate high-risk radiologic features such as contrast extravasation, large hemoperitoneum, or vascular blush on computed tomography [5].

SAE achieves hemostasis by reducing splenic arterial inflow while preserving parenchymal viability through collateral circulation from the short gastric and other vessels. Proximal embolization reduces overall splenic perfusion pressure, whereas distal (selective) embolization directly targets bleeding arterial branches. Combined techniques may be employed in complex vascular injuries. Although both techniques are widely practiced, controversy remains regarding optimal patient selection, comparative complication profiles including splenic infarction and abscess formation and long-term preservation of splenic immune function. Importantly, the current body of evidence is largely composed of retrospective cohort studies, and high-quality randomized comparative data remain limited. From an epidemiological standpoint, splenic injury accounts for approximately 25-40% of blunt abdominal trauma cases, with an estimated annual incidence of 10-15 per 100,000 population in high-income countries [6]. Modern trauma registry data demonstrate non-operative success rates exceeding 95% for low-grade injuries [7]. In high-grade (AAST III-V) injuries, incorporation of SAE into NOM protocols has improved splenic salvage rates to above 90% in many level I trauma centres [8]. Mortality directly attributable to isolated splenic injury in hemodynamically stable patients remains low, typically under 5%, and is more commonly associated with concomitant polytrauma rather than isolated splenic pathology [9].

The primary aim of this systematic review was to evaluate the clinical effectiveness and role of SAE as an adjunct to NOM in adult patients with high-grade (AAST grade III-V) blunt splenic trauma. Specifically, the review assessed technical success, splenic salvage rates, re-bleeding events, complications, delayed splenectomy, and mortality outcomes associated with SAE.

## Review

Materials and methods

Search Strategy

This systematic review was conducted in accordance with the Preferred Reporting Items for Systematic Reviews and Meta-analyses (PRISMA 2020) guidelines [10]. A comprehensive electronic database search was performed on 5 February 2026 across PubMed, Embase, Scopus, and the Cochrane Library. The search strategy combined Medical Subject Headings (MeSH) and free-text terms using Boolean operators as follows: (“splenic artery embolization” OR “splenic angioembolization” OR “SAE”) AND (“blunt splenic trauma” OR “blunt splenic injury” OR “splenic injury”) AND (“AAST” OR “high-grade” OR “grade III” OR “grade IV” OR “grade V”) AND (“proximal embolization” OR “distal embolization” OR “selective embolization”). Search filters were applied to include human studies involving adult populations. No initial language restrictions were applied; however, only full-text articles available in English were included at the eligibility stage. Reference lists of included studies were manually screened to identify additional relevant publications. The search results were exported into a reference management software for duplicate removal prior to screening.

Eligibility Criteria

Eligibility criteria were predefined using the population, intervention, comparator, outcome (PICO) framework to ensure methodological clarity and consistency with the objectives of this review [11]. The population (P) included adult patients (≥18 years) presenting with blunt splenic trauma classified as the AAST grade III-V. The intervention (I) of interest was SAE, including proximal, distal (selective), or combined embolization techniques performed as part of NOM. The comparator (C) included comparisons between embolization techniques (proximal versus distal versus combined) where available, as well as observational cohorts without a direct comparator, provided that the outcomes of SAE were clearly reported. The predefined outcomes (O) included technical success, splenic salvage rate, re-bleeding, complications (including splenic infarction, abscess formation, and access-related events), delayed splenectomy, and mortality. Only original clinical studies, including prospective or retrospective cohort studies evaluating SAE in high-grade blunt splenic trauma and reporting at least one relevant clinical outcome, were eligible for inclusion. Studies were excluded if they were case reports, small case series with fewer than 10 patients, pediatric-only studies, animal studies, editorials, narrative reviews, conference abstracts without full-text data, or studies involving penetrating splenic trauma. These criteria were designed to ensure inclusion of clinically relevant adult high-grade injury cohorts and were consistent with the characteristics of the five cohort studies summarized in the primary data table.

Study Selection

Two independent reviewers screened titles and abstracts for relevance. Full-text articles were subsequently assessed for eligibility against predefined criteria. Disagreements were resolved through consensus discussion. Only studies meeting all PICO components were included in the final synthesis.

Data Extraction

Data extraction was performed independently using a standardized form. Extracted variables included study design, sample size, patient demographics, injury grade, hemodynamic status, embolization technique, technical success rate, splenic salvage rate, re-bleeding rate, complication profile, delayed splenectomy, and mortality. Where technique comparisons were available, subgroup outcomes were recorded.

Risk of Bias Assessment

Risk of bias was evaluated using the Risk Of Bias In Nonrandomized Studies-of Interventions (ROBINS-I) for cohort studies [12]. Domains assessed included selection bias, comparability of cohorts, outcome assessment, and adequacy of follow-up. Given that all included studies were observational cohorts, the overall level of evidence was considered level III. Most studies demonstrated a moderate risk of bias due to retrospective design, absence of randomization, and limited long-term follow-up.

Data Synthesis

Due to heterogeneity in study design, patient populations, embolization techniques, and outcome reporting among the included studies, a quantitative meta-analysis was not considered appropriate. Therefore, a qualitative narrative synthesis was performed. The synthesis focused on key clinical outcomes, including technical success, splenic salvage rates, re-bleeding events, complications, delayed splenectomy, and mortality. Where available, outcomes were compared across proximal, distal, and combined embolization techniques. Patterns and trends across studies were summarized to identify similarities and differences in reported outcomes. Findings were interpreted in the context of methodological limitations and potential sources of bias within the included studies.

Protocol Registration 

This systematic review was not registered with a formal protocol registry (e.g., PROSPERO). However, the review was conducted in strict adherence to the PRISMA 2020 guidelines to ensure methodological rigor, transparency, and reproducibility. All key steps, including database selection, search strategy, inclusion and exclusion criteria, study selection, data extraction, and risk of bias assessment, were predefined and systematically documented prior to initiation of the review. Any deviations from the planned methodology have been clearly described in the manuscript.

Results

Study Selection Process

**Figure 1:**
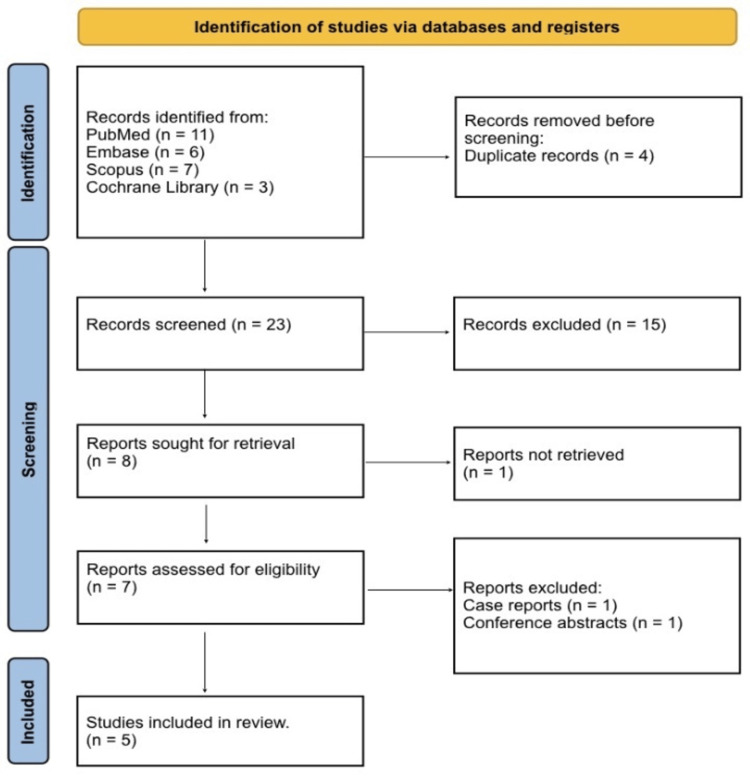
PRISMA 2020 flow diagram PRISMA: Preferred Reporting Items for Systematic Reviews and Meta-analyses

Figure 1 shows that a total of 27 records were identified from database searching. Of these, records were distributed across databases as follows: PubMed (n = 11), Embase (n = 6), Scopus (n = 7), and Cochrane Library (n = 3). After removal of duplicate records (n = 4), 23 studies remained for title and abstract screening. A total of 15 records were excluded at the screening stage due to irrelevance to high-grade injuries or absence of SAE-specific data. Eight reports were sought for full-text retrieval, of which one report was not retrievable. Seven full-text articles were assessed for eligibility. Two studies were excluded at the full-text stage: case reports (n = 1) and conference abstracts without complete outcome reporting (n = 1). No animal studies or editorials met the full-text eligibility stage after initial screening. Ultimately, five studies met all eligibility criteria and were included in the qualitative synthesis.

Characteristics of the Selected Studies

Five retrospective cohort studies comprising 600 adult patients with high-grade (AAST III-V) blunt splenic trauma were included (Table 1). Lin et al. [13] evaluated 202 hemodynamically stable patients undergoing proximal, distal, or combined SAE and reported 92.6% technical success and 95.5% splenic salvage with no significant difference between techniques. Clements et al. [14] analyzed 232 patients treated with mixed SAE, demonstrating 100% technical success, 97% salvage, 3.9% re-bleeding, and 3% delayed splenectomy. Nguyen et al. [15] reported 100% technical success and splenic preservation in 50 patients with no re-bleeding or major complications. Gill et al. reviewed 72 patients and observed a 90.3% salvage rate, with higher abscess formation (25%) in the combined technique group. Brahmbhatt et al. compared proximal versus distal embolization in 44 patients, noting re-bleeding requiring splenectomy in the distal group and higher fluoroscopy time with distal embolization (p = 0.004). Overall, SAE demonstrated high technical success and splenic salvage in stable high-grade injuries, with technique-related differences in complication profiles. Furthermore, mortality outcomes were inconsistently reported across the included studies and, therefore, could not be reliably synthesized; where not explicitly stated in the primary studies, mortality was recorded as “not reported."

**Table 1:** Clinical characteristics and outcomes of SAE in high-grade (AAST III-V) blunt splenic trauma AAST: American Association for the Surgery of Trauma; SAE: splenic artery embolization; PICO: population, intervention, comparator, outcome; MDCT: multidetector computed tomography

Study	Design (n)	Population	Intervention	Comparator	Outcomes	AAST grade	Hemodynamic status	Technical success	Splenic salvage	Re-bleeding	Major complications	Delayed splenectomy	Mortality	Key clinical findings
Lin et al., 2023 [13]	Retrospective cohort (n = 202)	Adults with blunt splenic injury undergoing angiography	SAE (proximal 31.7%, distal 41.6%, combined 26.7%)	Comparison between embolization techniques	Technical and clinical success, complications	III-V	Hemodynamically stable	92.6% overall	95.50%	Not separately reported	No significant difference between techniques	Not separately reported	Not reported	Comparable outcomes across proximal, distal, and combined techniques
Clements et al., 2020 [14]	Retrospective cohort (n = 232)	Adults with blunt splenic trauma treated with SAE	Mixed proximal + selective SAE	No direct surgical comparator	Clinical success, re-bleeding, complications	III-V (median IV)	Stable	100%	97%	3.90%	Infarction 1.3%; access complication 0.4%	3%	Not reported	High salvage rates; occult vascular lesions identified on angiography
Nguyen et al., 2023 [15]	Observational cohort (n = 50)	Adults with CT-confirmed vascular splenic injury	SAE (mixed technique)	No comparator	Technical success, splenic preservation	III-V	Stable	100%	100%	0%	None reported	0%	Not reported	Excellent short-term outcomes in MDCT-guided SAE cohort
Gill et al., 2023 [16]	Retrospective cohort (n = 72)	Adults with high-grade splenic injury	SAE (proximal, distal, combined)	Technique-based comparison	Salvage rate, complication profile	III-V	Stable	Not separately reported	90.30%	Not separately reported	Abscess 25% in the combined group	Not separately reported	Not reported	Combined technique associated with higher infectious complications
Brahmbhatt et al., 2021 [17]	Retrospective cohort (n = 44)	Adults with high-grade blunt splenic trauma undergoing SAE	Proximal SAE (n = 17)	Distal SAE (n = 23)	Complication rate, re-bleeding	III-V	STABLE	Not separately differentiated	Not separately reported	Re-bleeding in the distal group	Major complications: Prox 29.4%, Distal 21.7%	Splenectomy is required in a distal re-bleed	Not reported	Distal embolization associated with longer fluoroscopy time (p = 0.004)

Risk of Bias Assessment

Risk of bias assessment demonstrated variability across the included observational studies (Table 2). Lin et al. [13] showed a serious risk of bias due to confounding, moderate selection bias, and moderate risk of selective reporting, resulting in an overall serious risk of bias. Clements et al. [14] had a moderate risk related to confounding and selection bias, with low risk across other domains, leading to an overall moderate risk of bias. Nguyen et al. [15] demonstrated serious confounding bias and moderate selection and selective reporting bias, giving an overall serious risk. Gill et al. similarly had serious confounding bias, moderate selection bias, and moderate selective reporting bias, resulting in an overall serious risk [16]. Brahmbhatt et al. showed serious bias due to both confounding and selection, along with moderate selective reporting concerns, leading to an overall serious-to-high risk of bias [17]. Across studies, intervention classification, deviations from intended intervention, missing data, and outcome measurement were consistently assessed as low risk.

**Table 2:** Risk of bias assessment of included studies using ROBINS-I tool ROBINS-I: Risk Of Bias In Nonrandomized Studies-of Interventions Adapted/reproduced from Thomson et al. [12], with permission

Study	Bias due to confounding	Bias in selection of participants	Bias in classification of interventions	Bias due to deviations from intended interventions	Bias due to missing data	Bias in measurement of outcomes	Bias in selection of reported result	Overall risk of bias
Lin et al., 2023 [13]	Serious	Moderate	Low	Low	Low	Low	Moderate	Serious
Clements et al., 2020 [14]	Moderate	Moderate	Low	Low	Low	Low	Low	Moderate
Nguyen et al., 2023 [15]	Serious	Moderate	Low	Low	Low	Low	Moderate	Serious
Gill et al., 2023 [16]	Serious	Moderate	Low	Low	Low	Low	Moderate	Serious
Brahmbhatt et al., 2021 [17]	Serious	Serious	Low	Low	Low	Low	Moderate	Serious

Discussion

This systematic review demonstrates that SAE plays a pivotal role in the NOM of high-grade (AAST III-V) blunt splenic trauma. Across the included cohort studies, SAE consistently achieved high technical success rates (92-100%) and splenic salvage rates exceeding 90% in hemodynamically stable patients [13,17]. These findings align with modern trauma guidelines, including those from the World Society of Emergency Surgery (WSES), which advocate adjunctive angioembolization in stable patients with high-risk radiologic features [9]. Compared with historical splenectomy-based management, SAE preserves immunologic function while maintaining low mortality rates [5,8]. Importantly, failure of NOM in high-grade injuries appears substantially reduced when SAE is incorporated into standardized trauma protocols [7,14].

When comparing embolization techniques, proximal and distal approaches demonstrated broadly comparable splenic salvage rates in most studies, particularly in Lin et al. [13] and Clements et al. [14]. However, technique-related differences were observed in complication profiles. Distal embolization was associated with longer fluoroscopy times and occasional re-bleeding requiring splenectomy, whereas combined techniques showed higher rates of splenic abscess formation in Gill et al. Proximal embolization, by reducing overall splenic perfusion pressure while maintaining collateral flow, may theoretically reduce extensive infarction risk, although evidence remains observational [13,15,16]. Short-term outcomes across studies were excellent, with low re-bleeding and delayed splenectomy rates [14,15]. Long-term data, however, remain limited, particularly regarding immune preservation, risk of overwhelming post-splenectomy infection (OPSI), and quality-of-life outcomes.

Complication rates were generally low but not negligible. Reported adverse events included splenic infarction, abscess formation, access-site complications, and rare re-bleeding episodes [13]. Infectious complications appeared more frequently in combined embolization strategies, possibly due to greater parenchymal ischemia. Despite these risks, overall mortality directly attributable to splenic injury remained low and was more often related to concomitant polytrauma rather than embolization failure [8,9]. Importantly, most included studies lacked standardized definitions for complications and follow-up duration, limiting robust comparison across cohorts [17].

This review has several limitations. All included studies were retrospective observational cohorts with moderate to serious risk of bias, particularly due to confounding and selection bias. There were no randomized controlled trials directly comparing proximal, distal, and combined techniques. Heterogeneity in injury grading, embolization indications, operator expertise, and reporting standards precluded meta-analysis. Future research should focus on prospective multicentre registries and randomized comparative trials to clarify optimal technique selection, long-term immunologic outcomes, and cost-effectiveness. Standardized reporting frameworks and longer follow-up periods are essential to strengthen the evidence base and refine patient selection algorithms for SAE in high-grade splenic trauma.

## Conclusions

SAE appears to be a safe adjunct to NOM in hemodynamically stable patients with high-grade (AAST III-V) blunt splenic trauma, with reported studies showing high technical success and splenic salvage rates. Both proximal and distal embolization techniques are effective, though complication profiles may differ, and combined embolization may be associated with higher infectious risks. These conclusions are based on retrospective observational studies, and long-term outcomes, including immune function preservation, remain incompletely characterized. Future prospective multicentre studies are needed to optimize embolization strategies, standardize reporting, and evaluate both immunologic and quality-of-life outcomes.
